# Molecular epidemiology of drug-resistant *Plasmodium falciparum* in Benguela province, Angola

**DOI:** 10.1186/s12936-015-0634-2

**Published:** 2015-03-14

**Authors:** Vincent Foumane Ngane, Joseph Allico Djaman, Cécile Culeux, Nathalie Piette, Pierre Carnevale, Patrick Besnard, Filomeno Fortes, Leonardo K Basco, Rachida Tahar

**Affiliations:** Laboratoire de Recherche sur le Paludisme, Organisation de Coordination pour la lutte contre les Endémies en Afrique Centrale (OCEAC), B. P. 288, Yaoundé, Cameroon; Laboratoire de Pharmacodynamie Biochimique, Unité de Formation et de Recherche (UFR) Biosciences, Université Félix Houphouët-Boigny (Cocody), 22 BP 582, Abidjan, 22 Côte d’Ivoire; Département de Biochimie, Institut Pasteur de Côte d’Ivoire, 01 BP 490, Abidjan, 01 Côte d’Ivoire; Unité Mixte de Recherche 216 Mère et Enfant Face aux Infections Tropicales, Institut de Recherche pour le Développement (IRD), Unité de Formation et de Recherche (UFR) de Pharmacie, Université Paris Descartes, 4 avenue de l’Observatoire, 75270 Paris, France; Malaria Control Programme, Société nationale de métallurgie (Sonamet), rua 1 de Dezembro, caixa postal 479, Lobito, Provincia de Benguela Angola; Plano National Contra Malaria, Ministry of Health, Luanda, Angola; Unité de Recherche 198-Institut de Recherche pour le Développement (IRD), Unité de Recherche sur les Maladies Infectieuses et Tropicales Emergentes (URMITE), Faculté de Médecine La Timone, Aix-Marseille Université, 27 boulevard Jean Moulin, 13385 Marseille, France

**Keywords:** *Plasmodium falciparum*, Drug resistance, Antifolate drugs, Chloroquine, Molecular markers

## Abstract

**Background:**

The malaria situation has been worsening in Angola, partly due to armed conflict until the recent past and drug-resistant *Plasmodium falciparum*. Malaria transmission is heterogeneous within the country, and data on drug-resistant malaria in different parts of the country are incomplete. The aim of the present study was to evaluate resistance to 4-aminoquinolines and antifolate drugs in *P. falciparum* isolates collected in Benguela province, central Angola, using molecular markers.

**Methods:**

Fingerprick capillary blood was collected from asymptomatic children aged less than 15 years old during a household survey in and around Balombo town in 2010–2011. Samples were screened for *P. falciparum* by nested PCR. Molecular markers (*P. falciparum* dihydrofolate reductase [*pfdhfr*], *P. falciparum* dihydropteroate synthase [*pfdhps*], *P. falciparum* chloroquine resistance transporter [*pfcrt*], and *P. falciparum* multidrug-resistance gene 1 [*pfmdr1*]) were sequenced to determine the key codons associated with drug resistance.

**Results:**

A total of 60 blood samples were positive for *P. falciparum*. Most isolates with successful PCR amplification had mutant *pfdhfr* alleles, with either double mutant AICNI (69%) or triple mutant AIRNI (21%) haplotypes. A16V, S108T, and I164L substitutions were not found. Many of the isolates were carriers of either SGKAA (60%) or AGKAA (27%) *pfdhps* haplotype. K540E substitution was absent. There were only two *pfcrt* haplotypes: wild-type CVMNK (11%) and mutant CVIET (89%). Wild-type *pfmdr1* NYSND haplotype was found in 19% of the isolates, whereas single mutant *pfmdr1* YYSND and NFSND haplotypes occurred in 48% and 11%, respectively. Double mutant *pfmdr1* haplotypes (YFSND and YYSNY) occurred rarely.

**Conclusions:**

The results suggest that the high prevalence of mutant *pfcrt* CVIET haplotype is in agreement with low clinical efficacy of chloroquine observed in earlier studies and that the double *pfdhfr* mutant A**I**C**N**I and single *pfdhps* mutant SGKAA are currently the predominant haplotypes associated with antifolate resistance in Benguela province. The hallmark of clinical resistance observed in East Africa, i.e. triple *pfdhfr* mutant haplotype (AIRNI) and double *pfdhps* mutant haplotype (SGEAA), was absent. These molecular findings need to be further evaluated in parallel with clinical studies, in particular with the efficacy of intermittent preventive treatment using sulphadoxine-pyrimethamine in pregnant women and artesunate-amodiaquine for uncomplicated malaria.

## Background

Malaria is one of the major causes of morbidity and mortality in Angola. It is responsible for 35% of medical consultations, 20% of hospitalizations, and 25% of maternal deaths [1,2]. In 2010, the Angolan Ministry of Health reported that, of the estimated total population of 18–20 million, 3.7 million cases of malaria occurred, mostly due to *Plasmodium falciparum* [2].

Malaria is endemic throughout the country, but the transmission pattern is heterogeneous, varying from intense transmission and hyperendemicity in northern Angola to seasonal or epidemic, unstable malaria in the southern part of the country. In central Angola, including Benguela province, malaria transmission is characterized as meso-endemic and stable [2].

One of the causes of the worsening malaria situation in Angola was the civil war that ravaged the country and paralyzed the health system for three decades, until 2002. Another probable cause is drug-resistant malaria. The first chloroquine-resistant cases in the country were reported in non-immune expatriates returning to Europe from Angola in 1984 [3,4]. Low to moderate therapeutic efficacies of chloroquine, sulphadoxine-pyrimethamine, and amodiaquine monotherapies were confirmed in central Angola in 2002 [5]. Artemisinin-based combination therapy (ACT), using either artesunate-amodiaquine, artesunate-sulphadoxine-pyrimethamine, or artemether-lumefantrine, is highly effective [5,6]. Since 2006, the first-line drug for the treatment of uncomplicated malaria in Angola is artemether-lumefantrine, with artesunate-amodiaquine as an alternative drug combination.

In the face of challenges posed by the malaria situation, several clinical and epidemiological studies have been undertaken in recent years, including malaria indicator surveys covering the entire Angolan territory [2,7]. However, many of these recent studies have been conducted in Luanda, the capital city, its neighbouring provinces, or in the northern provinces [8-14]. Data from Benguela province are scarce. The aim of the present study was to analyse the epidemiology of drug-resistant *P. falciparum* isolates using molecular markers in several localities in and near the town of Balombo.

## Methods

### Study sites

**Figure 1:**
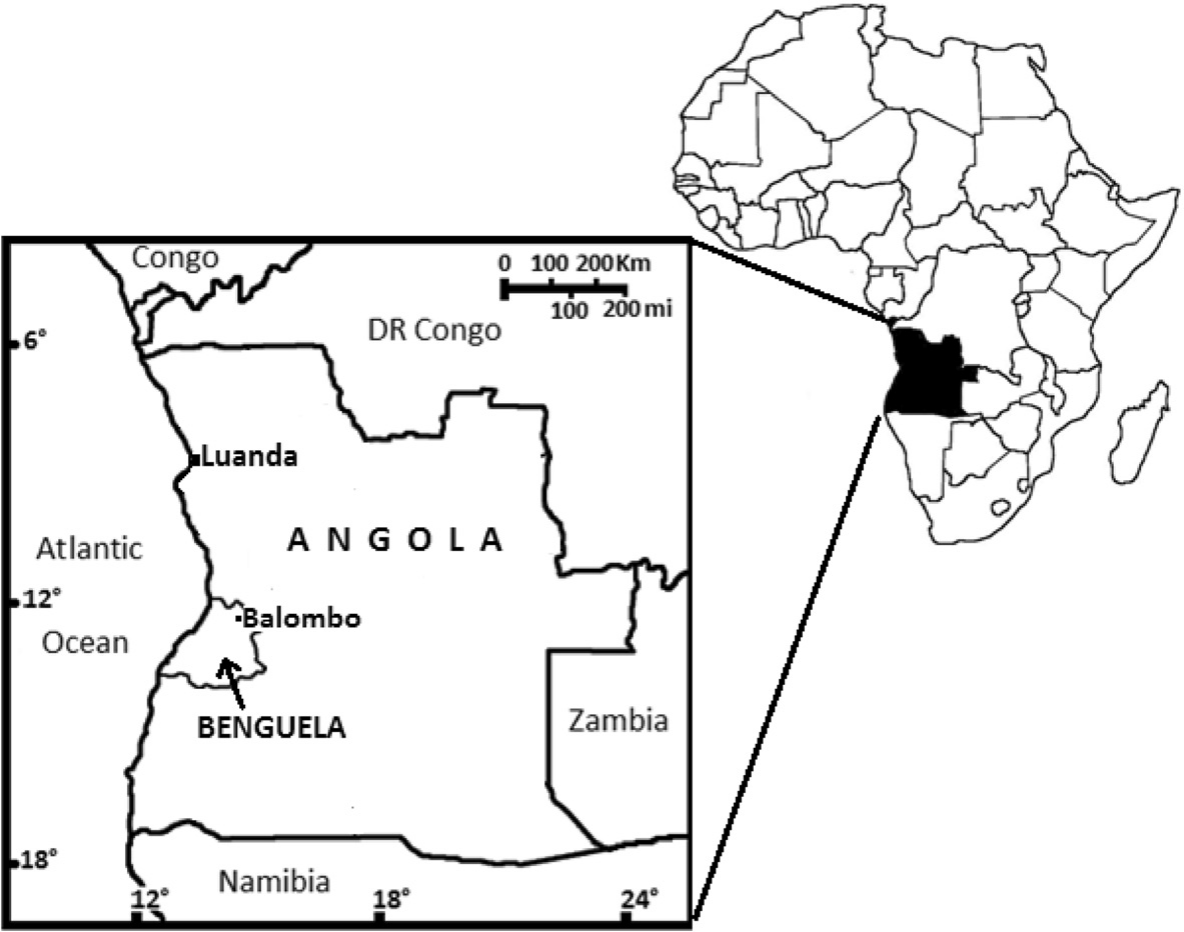
**Map of Angola showing Benguela province and Balombo.**

The study was conducted in 2010–2011 in the town of Balombo and its surrounding villages, situated in Benguela province in central Angola (Figure 1). Balombo (12°21’26” south, 14°46’16” east) is located about 150 km east of the coastal town of Lobito (12°19’11” south, 13°35’59” east) and 420 km to the south of Luanda (8°50’17” south, 13°14’3” east), the capital city of Angola. The estimated total population of Balombo and its surrounding villages was 48,465 inhabitants. The town is located in a valley surrounded by hills.

Benguela region has an equatorial climate characterized by a short dry season (June–September) and a long rainy season (October–May), with an average annual rainfall of 600–700 mm. Its geography is composed of savannah forests, with areas along the rivers covered by tropical forest. The major malaria vector in Benguela province is *Anopheles gambiae s.l.* [15,16].

Medical records in Balombo hospital indicate that presumptive malaria accounted for 46% of consultations and 62% of hospitalizations in 2005. However, since most malaria cases are diagnosed on a presumptive basis, the true prevalence of malaria is unknown in areas around Balombo. Nonetheless, a recent study has shown that malaria is highly prevalent in Benguela province and that severe and complicated malaria occurs in both children and adults [17].

### Asymptomatic *Plasmodium falciparum* carriers

The present study was part of household surveys conducted during the rainy season by the joint teams of Malaria Control Programme of Société nationale de métallurgie (Sonamet) and Services Nationaux de Santé Publique (Angolan Ministry of Health) of Benguela province. Households were selected by cluster sampling to obtain 30 representative households in the study sites. A number was assigned to the households, which were randomly selected using a table of random numbers. After obtaining a written informed consent signed by the head of each household on behalf of all household members, fingerprick capillary blood was obtained from the target group, i.e. children aged less than 15 years old, to prepare thick smears and store blood on Whatman 3MM filter paper. In this household survey, clinical examination was not performed, and the enrolled children were asymptomatic. Blood smears were immediately stained using 10% Giemsa and examined in the field. Parasites were counted against 50 white blood cells for rapid screening in the field. As this household survey on asymptomatic children was part of a training session of inexperienced Angolan laboratory technicians, the gold standard for malaria diagnosis was PCR in this study. If a child was smear-positive and presented fever or other signs and symptoms suggestive of malaria, he or she was referred to the nearest health centre for further examination and appropriate treatment.

### Ethical approval

This study was reviewed and approved by the National Malaria Control Programme of the Angolan Ministry of Health, the ethical authority that approves clinical studies on malaria research in Angola.

### DNA extraction and PCR

DNA was extracted from Whatman 3MM filter papers using Chelex beads [18]. Briefly, a 2 mm diameter disc was cut from Whatman filter paper and incubated at 4°C overnight in 0.5 mL of phosphate-buffered saline (PBS) containing 0.5% saponin. The filter paper was rinsed twice in saponin-free PBS, placed in 100 μL of distilled water containing 10% Chelex, and incubated at 100°C for 20 min to elute DNA. The supernatant containing extracted DNA was conserved at -20°C. A diagnostic nested PCR was performed using 18S rRNA as the target gene to detect samples with *P. falciparum* [19].

Molecular markers for drug resistance analysed in the present study (*P. falciparum* dihydrofolate reductase [*pfdhfr*], *P. falciparum* dihydropteroate synthase [*pfdhps*], *P. falciparum* chloroquine resistance transporter [*pfcrt*], and *P. falciparum* multidrug-resistance gene 1 [*pfmdr1*]) were amplified by nested PCR, as described in previous studies [20-22]. PCR products were sequenced using the Sanger dideoxy method. Nucleotide sequences were analysed using Chromas 2.4.1 software for DNA sequencing (Technelysium Pty Ltd., South Brisbane, Australia) and aligned using MultAlin software to identify mutations [23].

### Data interpretation and statistical calculations

A total of 576 children aged less than 15 years old were enrolled in the present household survey. The sample size was calculated on the basis of an expected smear-positive rate of 25% determined in an earlier pilot study in Balombo, risk of error of 5%, and 5% precision.

The sequences of the following key codons were determined: Ala16Val, Asn51Ile, Cys59Arg, Ser108Asn or Ser108Thr, and Ile164Leu for *pfdhfr*; Ser436Ala, Ala437Gly, Lys540Glu, Ala581Gly, and Ala613Thr or Ala613Ser for *pfdhps*; codons Cys72Ser, Val73 (invariable), Met74Ile, Asn75Glu, Lys76Thr for *pfcrt*; and Asn86Tyr, Tyr184Phe, Ser1034Cys, Asn1042Asp, and Asp1246Tyr for *pfmdr1*. Based on these codons, different haplotypes were determined and compared. Wild-type *pfdhfr* haplotype was defined as ANCSI. Double *pfdhfr* mutant alleles were defined as Ile-51/Asn-108 or Arg-59/Asn-108 (A**I**C**N**I or AN**RN**I haplotypes). Triple *pfdhfr* mutant alleles were defined as Ile-51/Arg-59/Asn-108 (A**IRN**I haplotype). Wild-type *pfdhps* refers to SAKAA haplotype. *Pfcrt* haplotype was defined as wild-type CVMNK and chloroquine-resistant type CVIET. Wild-type *pfmdr1* haplotype denotes NYSND.

## Results and discussion

A total of 386 blood samples collected in and around Balombo town were screened for the presence of malaria parasites by microscopy and nested PCR. Sixty-one and 80 samples were positive for *P. falciparum* by microscopy and diagnostic nested PCR, respectively. Sixty PCR-positive samples were further analysed to determine the key codons of *pfdhfr*, *pfdhps*, *pfcrt*, and *pfmdr1* genes.

Fifty-eight of 60 samples were successfully amplified to determine *pfdhfr* alleles. A large majority (69%) carried double Ile-51/Asn-108 mutations, while only 3 (5.2%) had the alternative Arg-59/Asn-108 double mutations (Table 1). Triple *pfdhfr* mutant alleles (AIRNI haplotype) were present in 20.7% of the field isolates. Both wild-type (n = 2; 3.4%) and single Asn-108 mutant allele (n = 1; 1.7%) were rarely observed. All isolates had the wild-type Ile-164 allele.

**Table 1:** ***pfdhfr*** **,** ***pfdhps*** **,** ***pfcrt*** **, and** ***pfmdr1*** **haplotypes of** ***Plasmodium falciparum*** **isolates in Balombo area, Benguela province, central Angola**

**Molecular marker (no. of isolates analysed)**	**No. of isolates (%)**
*dhfr* (n = 58)	
Wild-type haplotype ANCSI	2 (3.4)
Single mutant haplotype ANC**N**I	1 (1.7)
Double mutant haplotype A**I**C**N**I	40 (69.0)
Double mutant haplotype AN**RN**I	3 (5.2)
Triple mutant haplotype A**IRN**I	12 (20.7)
*dhps* (n = 30)	
Wild-type haplotype SAKAA	4 (13.3)
Single mutant haplotype S**G**KAA	18 (60.0)
Double mutant haplotype **AG**KAA	8 (26.7)
*pfcrt* (n = 36)	
Wild-type haplotype CVMNK	4 (11.1)
Mutant haplotype CVIET	32 (88.9)
*pfmdr1* (n = 54)	
Wild-type haplotype, NYSND	19 (35.2)
Single mutant haplotype, **Y**YSND	26 (48.1)
Single mutant haplotype, N**F**SND	6 (11.1)
Double mutant haplotype, **YF**SND	2 (3.7)
Double mutant haplotype, **Y**YSN**Y**	1 (1.8)

For each haplotype, mutant alleles are in bold.

In a study on 21 isolates collected in Huambo, a neighbouring province to the east of Benguela province, all carried the mutant Asn-108 *pfdhfr* allele, and the double mutant haplotype Ile-51/Asn-108 was the most prevalent [24]. Overall, in that study, 25% of the isolates collected from different localities in the country presented the triple *pfdhfr* mutant alleles. In another study on isolates collected in Luanda [25], 31 of 61 (51%) samples were triple *pfdhfr* mutants, and 25 of 61 (41%) and 4 of 61 (6.6%) isolates were Ile-51/Asn-108 and Arg-59/Asn-108 double mutants, respectively. In the northern province of Uige, the majority (40 of 66, 61%) of clinical isolates were double Ile-51/Asn-108 *pfdhfr* mutants, followed by triple mutants (23 of 66, 35%) and the alternative double Arg-59/Asn-108 mutant (n = 1) and wild-type (n = 2) [26]. The results of several recent studies, including those of the present study, suggest that the double *pfdhfr* mutant A**I**C**N**I is currently the predominant haplotype in several regions in Angola, with the possible exception of Luanda where triple *pfdhfr* mutants seem to predominate.

Thirty isolates were randomly selected for sequencing of *pfdhps* alleles. Most (60%) were characterized by the single mutant Gly-437, i.e. SGKAA haplotype (Table 1). The double mutant haplotype AGKAA and wild-type SAKAA were found in 8 (26.7%) and 4 (13.3%) isolates, respectively. Mutations were not observed in codons 540, 581, or 613.

Similarly, in Luanda, SGKAA was also the most frequent haplotype (18 of 30, 60%), followed by AGKAA (7 of 30, 23%) and the wild-type SAKAA (2 of 30, 7%) [25]. SGEAA and SGEGA haplotypes were found in 3 isolates in that study. The presence of Glu-540 *pfdhps* allele at a low frequency (14 of 221 samples, 6.3%) was also reported from an earlier study in Luanda [27]. In a study on *P. falciparum* isolates from different parts of the country [24], SGKAA haplotype largely predominated over other *pfdhps* haplotypes (339 of 372, 91%). In that study, there were 5 isolates with SGKAA, 1 SGEAA, and 2 SAKAA among 8 isolates collected from Huambo, the neighbouring province of Benguela. The wild-type SAKAA (21 of 372, 5.6%) and double mutant haplotype SGEAA (12 of 372, 3.2%) were found at low frequencies in different regions of Angola. In the northern province, most isolates were characterized by AGKAA (34 of 66, 51.5%), SGKAA (27 of 66, 40.9%), and SAKAA (5 of 66, 7.6%) [26]. Taken together, SGKAA is the major *pfdhps* haplotype in Angola, including Benguela province, and there appears to be a regional variation in the frequency of AGKAA haplotype. Both Glu-540 mutant allele and the wild-type SAKAA haplotype occur at low frequencies. These molecular data need to be interpreted in the light of clinical efficacy of sulphadoxine-pyrimethamine for intermittent preventive treatment in pregnancy.

Among 36 isolates randomly selected for PCR amplification and sequencing of *pfcrt*, a large majority (88.9%) were characterized by the mutant haplotype CVIET. The other isolates were of wild-type haplotype CVMNK. Ser-72 was not found in the present study.

In one of the studies conducted in Luanda in which only codon 76 was determined, Thr-76 was found in 230 of 241 (94%) isolates [27]. In the second study in Luanda, the mutant *pfcrt* haplotypes included the South American-type SVMNT (58 of 102, 57%), CVIET (13 of 102, 13%), and minor and mixed haplotypes [28]. The *pfcrt* SVMNT haplotype has been rarely reported from the African continent. This haplotype is more commonly found in South America and in some Asian countries and is associated with resistance to amodiaquine and chloroquine [29]. It has been hypothesized that the unusual predominance of SVMNT in Luanda may possibly be due to frequent travels and commercial exchanges between the Angolan capital city and Brazil [28]. However, other molecular studies conducted elsewhere in Angola, including the present study, have not reported the presence of SVMNT haplotype. In Bengo and Uige provinces in the north, only two *pfcrt* haplotypes were observed: CVIET (273 of 430, including mixed haplotypes, 63.5% in Bengo; 62 of 66 isolates, 94% in Uige) and CVMNK (157 of 430, excluding mixed haplotypes, 36.5% in Bengo; 4 of 66, 6% in Uige) [26,30]. With the exception of SVMNT haplotype found in Luanda, the results of the present study, as well as those performed earlier in other provinces, indicate that most isolates are carriers of mutant CVIET haplotype and are in agreement with the high prevalence of chloroquine-resistant *P. falciparum* in Angola.

Of 54 isolates analysed in the present study, most (29 of 54, 53.7%) carried the mutant *pfmdr1* allele Tyr-86, but mutations occurred less frequently in codons 184 (8 of 54, 14.8%) and 1246 (1 of 54, 1.9%). All isolates had the wild-type Ser-1034 and Asn-1042 *pfmdr1* codons.

In an early study, of 15 Angolan *P. falciparum* isolates (11 chloroquine-resistant and 4 chloroquine-sensitive in vitro), the majority (73%) of chloroquine-resistant isolates carried the mutant Tyr-86 [31]. In Luanda, pure Tyr-86 allele and Tyr- and Asn-86 mixed alleles were found in 142 of 199 (71%) isolates [27]. In the northern province of Uige, many isolates (45 of 66, including mixed Asn- and Tyr-86; 68%) also had the mutant Tyr-86 [26]. However, in Bengo province, the pure wild-type Asn-86 was predominant (94 of 138, 68%), mutant Phe-184 (pure or mixed alleles) occurred in 54 of 138 isolates (39.1%), and codons 1034, 1042, and 1246 were all wild-type [30]. These studies indicate that the majority of Angolan isolates carry the mutant Tyr-86 *pfmdr1* allele.

Amodiaquine, in combination with artesunate, is widely used in sub-Saharan African countries. Previous studies have suggested that failure after amodiaquine treatment is associated with the selection of *P. falciparum* isolates carrying *pfcrt* Thr-76 allele and *pfmdr1* haplotype Tyr-86, Tyr-184, and Tyr-1246 [32-34]. In the present study, most isolates had the mutant *pfcrt* Thr-76 allele but the *pfmdr1* mutant haplotype associated with amodiaquine resistance was rare (1 of 54 isolates, 1.8%). These molecular findings suggest amodiaquine efficacy but require clinical validation studies in Benguela, Angola.

## Conclusions

The results of the present study suggest that the high prevalence of mutant *pfcrt* CVIET haplotype is in agreement with low clinical efficacy of chloroquine observed in an earlier study conducted in the neighbouring province [5]. However, the *pfmdr1* haplotype associated with amodiaquine failure was rare, suggesting amodiaquine efficacy. In Benguela province, the double *pfdhfr* mutant AICNI and single *pfdhps* mutant SGKAA are currently the predominant haplotypes associated with antifolate resistance. The hallmark of clinical resistance observed in East Africa, i.e. triple *pfdhfr* mutant haplotype (AIRNI) and double *pfdhps* mutant haplotype (SGEAA), was absent. These molecular findings need to be further evaluated in parallel with clinical studies, in particular with the efficacy of intermittent preventive treatment using sulphadoxine-pyrimethamine in pregnant women and therapeutic efficacy of artesunate-amodiaquine.

## Abbreviations

ACT: Artemisinin-based combination therapy
*pfdhfr*: *Plasmodium falciparum* dihydrofolate reductase
*pfdhps*: *Plasmodium falciparum* dihydropteroate synthase
*pfcrt*: *Plasmodium falciparum* chloroquine resistance transporter
*pfmdr1*: *Plasmodium falciparum* multidrug-resistance gene 1
